# Investigating Ex Vivo Animal Models to Test the Performance of Intravitreal Liposomal Drug Delivery Systems

**DOI:** 10.3390/pharmaceutics13071013

**Published:** 2021-07-02

**Authors:** Gustav Christensen, Leon Barut, Dileep Urimi, Nicolaas Schipper, François Paquet-Durand

**Affiliations:** 1 Institute for Ophthalmic Research, University of Tübingen, Elfriede-Aulhorn Straße 5-7, 72076 Tübingen, Germany; gustav.christensen@uni-tuebingen.de (G.C.); leon.barut@student.uni-tuebingen.de (L.B.); 2 Division Bioeconomy and Health, Chemical Process and Pharmaceutical Development, RISE Research Institutes of Sweden, Forskargatan 18, 151 36 Södertälje, Sweden; dileep.urimi@ri.se (D.U.); nicolaas.schipper@ri.se (N.S.)

**Keywords:** liposomes, intravitreal, ocular drug delivery, retinal explants

## Abstract

There is a strong need for innovative and efficient drug delivery systems for ocular therapy development. However, testing intravitreal drug delivery systems without using live animals is challenging. Ex vivo animal models offer an interesting alternative. We analyzed the potential of using fresh porcine eyes obtained from the local slaughterhouse as a model for testing the intravitreal biodistribution and retention of liposomes with or without polyethylene glycol (PEG) conjugation and with different surface charges. The histology of the eyes was analyzed to localize the liposomes, and it was found that liposomes with PEG absorbed rapidly on the retina (within 1 h), with positively charged and PEG-coated liposomes being retained for at least 24 h. In parallel, fluorophotometry was employed on intact eyes, to determine the pharmacokinetics of the fluorophore calcein, as a substitute for a small hydrophilic therapeutic compound. We found a 4.5-fold increase in the vitreous half-life of calcein loaded in liposomes, compared with the free solution. Retinal toxicity was addressed using murine-derived retinal explant cultures. Liposomes were non-toxic up to 500 µg/mL. Toxicity was observed at 5 mg/mL for anionic and cationic liposomes, with 2-fold and 2.5-fold increased photoreceptor cell death, respectively. Overall, we could show that important ocular drug delivery considerations such as pharmacokinetics and biodistribution can be estimated in ex vivo porcine eyes, and may guide subsequent in vivo experiments.

## 1. Introduction

Treating retinal diseases with injections into the vitreous body of the eye is common practice in the clinic. Repeated intravitreal administration is part of the treatment of certain diseases like age-related macular degeneration [1] and diabetic retinopathy. However, in addition to being unpleasant and a reason for patient incompliance, intravitreal injections can lead to complications, such as retinal detachment, vitreous hemorrhage, and endophthalmitis [2]. Encapsulation of drugs in drug delivery systems can extend the vitreous retention time of the drug, and reduce the injection frequency [3]. In this regard, nano-sized liposomes offer promising potential. They are composed of usually well-tolerated lipids, and less material is required to form the liposomes as compared to other colloidal drug delivery systems (such as lipid-nanoparticles and polymersomes), which restricts scattering of in-coming light through the vitreous [4]. Liposomes can encapsulate hydrophilic and hydrophobic compounds simultaneously and the surface can be easily modified, e.g., the surface charge can be fine-tuned or targeting ligands to certain cell types can be conjugated [4,5,6,7]. In addition, they are biodegradable, and potentially more safe for repeated applications than non-degradable drug delivery systems like metallic nanoparticles [5].

The vitreous is a gel-like substance and provides the structural integrity of the eyeball. It consists mostly of water (98–99%), whereas the dry materials include fibers (mainly collagen type II), hyalocytes, and sugars [8]. The high water content means that in particular small hydrophilic molecules (<1000 Da) diffuse rapidly through the vitreous with a half-life typically not more than a few hours [9,10,11,12,13]. In general, it is understood that liposomes with a positive surface potential diffuse slower in the vitreous than those with negative or neutral surface potentials [14], due to the presence of negatively charged components such as hyaluronic acid and heparan sulfate [8]. Grafting polyethylene glycol (PEG) polymers on the liposome surface is a common technique to diminish immune responses, otherwise induced by the nanocarriers [15]. This results in an improved circulating half-life of systemically administered PEGylated liposomes. In the vitreous, immunological activity is limited compared to the rest of the body, and there is evidence that PEG enhances the vitreous diffusion of liposomes by shielding electrostatic interaction of the liposomes with vitreal components [7,16,17]. This could mean that the vitreous retention time of PEGylated liposomes is less than their non-PEGylated counterpart.

Testing viable intravitreal drug delivery systems in preclinical studies is often restricted to in vivo experimentation in live animals [16,17], which is usually expensive, time-consuming, and carries ethical considerations. Moreover, often rabbits are used [18], although the volume of the rabbit vitreous is only around 1.5 mL, while it is 3–4 mL for humans [16]. Interestingly, an in vitro eye model (PK-Eye^TM^) has been developed to estimate human pharmacokinetics of intravitreal therapeutic compounds and formulations, which are cleared through the anterior route [13,19]. The model has similar dimensions to human eyes, and the rate of clearance between two compartments can be measured. However, the model does not address the tissue absorption of the injected formulations. Porcine eyes obtained from leftover carcasses at slaughterhouses could provide an alternative, as the porcine vitreous is large (4 mL), and histological sections can be prepared to localize fluorescently labelled drug delivery systems within the eyes [20]. Porcine ex vivo organs have previously been used as a surrogate for in vivo systems. For instance, cornea absorption of nanoparticles has been tested [21], and porcine skin has been used to obtain pharmacokinetic parameters of topical drug delivery systems [22].

Here, we investigated the potential of using ex vivo porcine eyes obtained from the slaughterhouse as a model for testing intravitreally administered drug delivery systems. Liposomes with positive, neutral, or negative surface potentials, with or without surface-grafted PEG were chosen as delivery systems, as the vitreous diffusion of similar formulations is available from the literature. Non-invasive ocular fluorophotometry was used to measure the spread of intravitreally injected liposomes loaded with a hydrophobic fluorophore. To further assess the tissue distribution within the eye, a full histological workup was performed and correlated with the non-invasive imaging data. The intravitreal pharmacokinetics were then evaluated for a hydrophilic fluorophore encapsulated in the most promising liposome formulation. Lastly, the potential retinal cytotoxicity of these liposomes was assessed using organotypic murine retinal explant cultures.

## 2. Materials and Methods

### 2.1. Materials

A total of 99% chloroform with 0.5–1% ethanol, 1-palmitoyl-2-oleoyl-sn-glycero-3-phosphocholine (POPC), cholesterol, 1,2-dioleoyl-3-trimethylammonium-propane (chloride salt) (DOTAP), 1,2-distearoyl-sn-glycero-3-phosphoethanolamine-*N*-[amino(polyethylene glycol)-2000] (ammonium salt) (DSPE-PEG_2000_), 1-palmitoyl-2-oleoyl-sn-glycero-3-phospho-L-serine (sodium salt) (POPS), dioctadecyl-3,3,3,3 Tetramethylindodicarbocyanine (DiO), calcein, sodium chloride, hydrogen chloride, sodium hydroxide, disodium hydrogen phosphate dihydrate, sodium dihydrogen phosphate monohydrate, paraformaldehyde, (4-(2-hydroxyethyl)-1-piperazineethanesulfonic acid) (HEPES), proteinase K, fetal bovine serum, polycarbonate membranes (0.1 µm pore size), and filter supports were obtained from Sigma Aldrich (Darmstadt, Germany). R16 medium and dialysis cassettes (Slide-A-Lyzer, cellulose, 100K molecular weight cut-off) were obtained from Thermo Fisher Scientific (Waltham, MA, USA).

### 2.2. Methods

#### 2.2.1. Preparation of Liposomes

Liposomes were produced using the thin-film hydration method [23]. All lipids were dissolved in chloroform and mixed in the respective molar ratios summarized below in Table 1 in a round-bottom flask. For DiO-labelled liposomes, 0.2 mol% DiO was added. The solvent was evaporated using a rotary evaporator (model RC600, KNF Neuberger, Trenton, NJ, USA) at 50 °C, 105 rpm and 300 mbar for at least 1 h, until a thin film of dried lipids was left. For samples characterized with dynamic light scattering, the film was hydrated in 10 mM HEPES buffer (pH 7, adjusted with NaOH). For other formulations, phosphate-buffered saline (PBS) was added. The lipid suspension was extruded with a mini-extruder (Avanti Polar Lipids, Alabaster, AL, USA) through a polycarbonate membrane with 100 nm pore sizes between two filter supports, for at least 11 times, and stored at 4 °C.

The liposome size and size distribution were measured using dynamic light scattering at a detection angle of 173°. Samples were analyzed in a disposable low volume cuvette at a sample refractive index of 1.47 at 25 °C. The ζ-potential of the samples was obtained by measuring the electrophoretic mobility of the particles under the influence of an applied electric field. Both size and ζ-potential measurements were carried out on Zetasizer Nano ZS (Malvern Instruments, Malvern, UK) using the Zetasizer Software version 7.13 (Malvern Instruments, Malvern, UK). Liposome concentrations of 0.3 mg/mL in 10 mM HEPES were used for the analysis.

#### 2.2.2. Distribution of Liposomes in Porcine Vitreous Assessed with Fluorophotometry

Fresh porcine eyes were obtained from the slaughterhouse. Before use, the eyes were washed briefly with PBS buffer. Background fluorescence was measured on an ocular fluorometer (FM-2 Fluorotron Master, OcuMetrics, CA, USA), which records green fluorescent signal along the axial length. Triplicate recordings were obtained for each measurement. A total of 50 µL liposomes encapsulating the fluorophore dioctadecyl-3,3,3,3 Tetramethylindodicarbocyanine (DiO) (12.5 mg/mL, PBS) was injected with a 22-gauge needle (Hamilton 1705 RN syringe, Hamilton, Reno, NV, USA) into the center of the eye (roughly 11 mm axially from the optic nerve). The signal from DiO-labelled liposomes was measured immediately after injection (t = 0). Subsequently, the eyes were submerged in PBS buffer and put on a platform shaker (Heidolph Instruments, Schwabach, Germany) at 45 rpm to simulate eye movement. This speed corresponds to the average or lower end of saccadic movements [24,25]. The signal was measured after 1, 2, 6, and 24 h. Eyes without liposome injection were used to determine the amount of auto-fluorescence.

#### 2.2.3. Histological Evaluation of Liposomes Intravitreally Injected in Porcine Eyes

After injection of DiO-loaded liposomes in porcine eyes and incubation for either 1 or 24 h, the eyes were fixed in 4% paraformaldehyde in PBS (PFA). Eyes that were not injected were used as control. The fixation protocol was adapted from a published protocol [20] with a few changes. Firstly, a 27-gauge needle was used to puncture 4 holes in roughly equal distance around the limbus. Secondly, 200 µL PFA was intravitreally injected just below the limbus. Eyes were submerged to PFA and left at 4 °C for 5 days. Afterwards, the eyes were washed twice in PBS for 10 min, transferred to an embedding medium (Tissue-Tek O.C.T. Compound, Sakura Finetek Europe, Alphen aan den Rijn, Netherlands) and snap frozen with liquid N_2_. The frozen eyes were sectioned on a cryostat (NX50, Thermo Fisher, Waltham, MA, USA) to produce 40 μm thick sections on Superfrost Plus^TM^ object slides (R. Langenbrinck, Emmendingen, Germany). The sections were dried for at least 1 h at 37 °C and rehydrated with PBS. Mounting medium with DAPI (Vectashield with DAPI, Vector laboratories, Burlingame, CA, USA) was added to the sections, and they were kept at 4 °C for at least 30 min prior to imaging with fluorescent microscopy (Axio Imager Z2 with ApoTome function, Zeiss, Oberkochen, Germany). A CCD camera with a 20× objective was used. Ex./Em. wavelengths of 483/501 nm were used to detect the green DiO signal. Image acquisition was done by recording 30 z-stacks 1 µm apart and projecting the images using the Maximum Intensity Projection function in the acquisition software (ZEN 2.6, Zeiss). The same software was used to measure the mean fluorescent signal from the green channel. For the analysis, 3 eyes were measured per condition (*n* = 3). For each condition, 10–15 z-stacks were recorded at different areas on the retina and around 10 z-stacks were obtained from the vitreous, using 3 different microscopy slides (30–45 images for the retina, and 30 images for the vitreous per condition). The green DiO signal from all images were averaged. The background signal was obtained from un-injected eyes. The average background value was subtracted from all the other values. One-way AVONA with Tukey’s post-hoc test was calculated with GraphPad Prism 8 (GraphPad Software, San Diego, CA, USA) to assess statistical significance within the dataset.

#### 2.2.4. Assessment of Pharmacokinetic Parameters

The most promising liposome formulation in the previous distribution experiment was chosen to encapsulate the hydrophilic fluorophore calcein (622.55 g/mol). Liposomes (25 mg/mL) with 2 mM calcein (pH 7, adjusted with NaOH) in PBS were prepared as described above, and excess calcein was removed with dialysis against 150 mM NaCl at a volume 2000X that of the sample. The dialysate was exchanged after 2 h, 4 h, and then left overnight. The liposomes were sterile-filtered (Millex-GP, 0.22 µm, polyethersulfon, Merck Milipore, Darmstadt, Germany) and the fluorescent intensity at 530 nm was measured on a microplate reader (Tecan Spark 10M, Tecan, Männedorf, Switzerland) to determine the concentration of encapsulated calcein with a standard curve (Supplemental data, Figure S1A). Before injection, the calcein concentration was diluted to 90 µM. The same concentration of free calcein was used as control. As the porcine eyes used in this experiment were intended for extended use (>24 h), they were cleaned with 70% ethanol, kept at sterile conditions, and only handled using sterile tools. The eyes were washed in 70% ethanol and sterile-filtered PBS every day. Excess tissue were dissected from the eyes before injection. A 27-gauge needle was used to inject 50 µL of the liposome/calcein or free calcein solution into the vitreous through the *pars plana* (roughly 5 mm from the limbus). The calcein signal was measured using fluorophotometry (FM-2 Fluorotron Master, OcuMetrics, CA, USA), and the signal across the vitreous was averaged. Only data points where the calcein signal was observed to be constant across the vitreous were used. This was done to account for a potential uneven distribution. A standard curve of free calcein in extracted porcine vitreous (Supplemental data, Figure S1B) was obtained and used to determine the calcein concentration in the intact porcine vitreous.

#### 2.2.5. Retina Viability Assay

##### Animals

The C3H wild-type mouse line was used in all studies [26]. Animals were used irrespective of gender and were housed with free access to food and water under standard white light with 12 h light/dark cycles. They were sacrificed at postnatal day 13 by CO_2_ asphyxiation, followed by cervical dislocation. All procedures were performed in accordance with §4 of the German law on animal protection and approved by the animal protection committee of the University of Tübingen (Einrichtung für Tierschutz, Tierärztlichen Dienst und Labortierkunde; Registration No. AK02/19M, 3. April 2019).

##### Explanation and Culturing of Mouse Retinas

Retinas from mice eyes were isolated following a previously published protocol [27]. First, the eyes were pretreated in proteinase K to dissolve layers around the retina. Incubation in 20% fetal bovine serum stopped the proteinase reaction. Then, they were dissected under sterile conditions to separate the retina together with its retinal pigment epithelium. The retina was cut in a four-leaf clover shape, so that it could be placed flatly in a Transwell polycarbonate membrane (0.4 µm, Corning-Costar, New York, NY, USA) with the epithelium facing the membrane. Serum-free R16 medium, which was free of antibiotics and contained essential nutrients for retinal cultures [28], was used. Medium was exchanged every second day. After two days, a 20 µL solution of sterile-filtered liposomes were added on top of the cultures and incubated with the retinas for 24 h, after which the retina cultures were fixed in PFA. For cryoprotection, the cultures were incubated at room temperature in rising concentrations of sucrose (10, 20, and 30%) in PBS for 10 min, 20 min, and overnight at 4 °C, respectively. The retinas were submerged in embedding medium and frozen with liquid N_2_. They were sectioned to produce 14 µm thick sections on Superfrost Plus^TM^ slides.

##### Assessing Cell Death in Retinal Sections Using the TUNEL Assay

To detect dying cells in the retina cultures after addition of liposomes, a terminal deoxynucleotidyl transferase dUTP nick end labeling (TUNEL) assay was used. The protocol is available elsewhere [29,30]. The detection kit TMR red, Product No. 12156792910 (Sigma Aldrich, Darmstadt, Germany) was used. Retinal explant cultures incubated with PBS buffer (control) or increasing concentrations of the liposome formulations An-PEG-Lp or Cat-PEG-Lp (see Table 1) were tested. DAPI was used as nuclear counterstain. Fluorescent microscopy with red (Ex./Em. 548/561 nm) and blue (Ex./Em. 353/465 nm) channels to detect the TUNEL-induced labelling of dying cells and DAPI staining, respectively, was used. A total of 12 z-stacks 1 µm apart were recorded, and the relative amount of TUNEL-positive cells were manually counted and averaged for each retinal culture (representing one animal). The results were analyzed with one-way ANOVA and Dunnett’s multiple comparisons test (α = 0.05).

## 3. Results

### 3.1. Liposome Characterization

To confirm the successful formation of liposomes, all formulations were analyzed with dynamic light scattering (Table 2). In this study, the effect of charge and PEGylation of the liposomes in the porcine vitreous distribution and retention was tested. Therefore, liposomes that were either anionic, cationic, or more neutrally charged were prepared either with or without surface-grafted PEG. The charge of each formulation was determined by measuring the ζ-potential.

Similar sizes were observed for all formulations with narrow size distributions, except for the more neutrally charged liposomes, which were slightly larger (20–30 nm) and shown more polydispersity. The Neu-PEG-Lp formulation were more positively charged than expected (around 29 mV), probably due to insufficient shielding of the positive charges by the PEG chains.

### 3.2. Liposome Distribution in Ex Vivo Porcine Eyes

**Figure 1:**
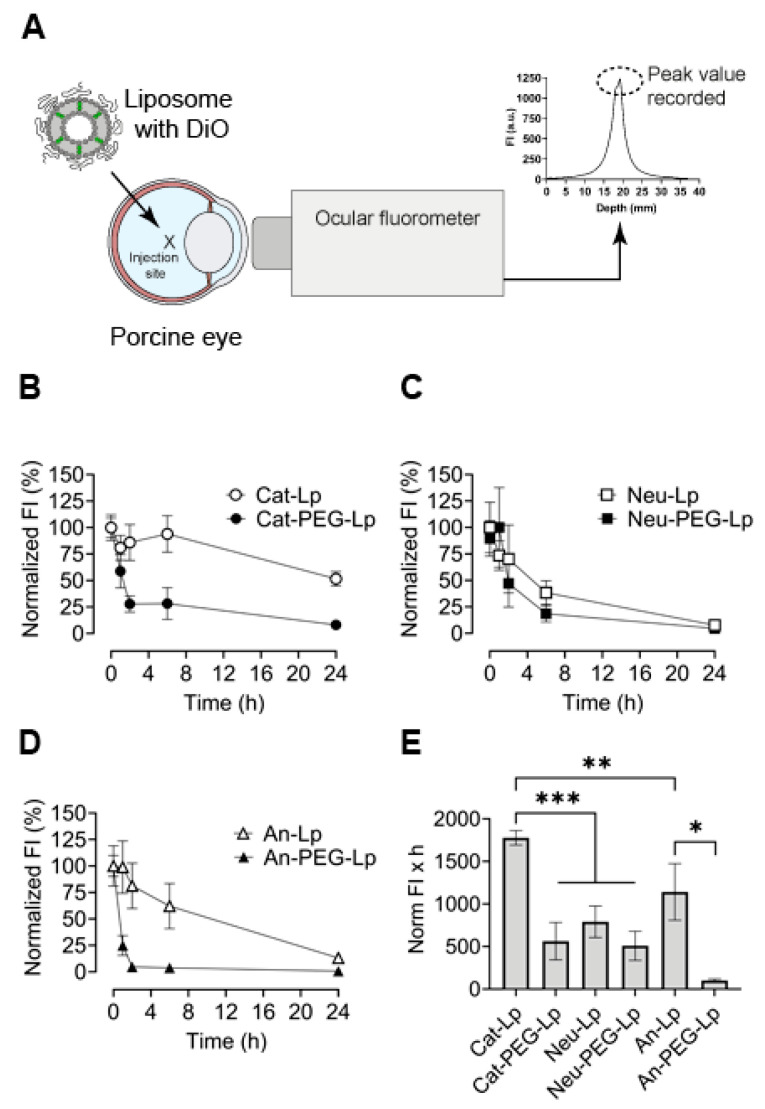
Diffusion dynamics of liposomes (Lp) intravitreally administered to intact ex vivo porcine eyes. (**A**) Liposomes were loaded with a hydrophobic green fluorophore (DiO), injected into the center of eyes. DiO signal was recorded non-invasively after specific time-points on an ocular fluorophotometer. (**B**–**D**) Peak signal was used to plot the normalized fluorescent intensity (FI) over time for cationic (Cat), neutrally charged (Neu), and anionic (An) formulations with or without poly(ethylene glycol) (PEG) grafted on the liposome surface. (**E**) The area under the normalized FI-time curves (**B**–**D**) was determined for each formulation. Results represent mean ± SEM for *n* = 4–6. * = *p* < 0.05, ** = *p* < 0.01, *** = *p* < 0.001. Statistical analysis: One-way ANOVA with Tukey‘s post-hoc test.

Fluorophotometry was used to analyze the vitreous distribution of liposome in intact ex vivo porcine eyes. The distribution kinetics of the formulations summarized in Table 1 were measured to identify comparative differences and establish an initial framework for further experiments. A green hydrophobic fluorophore (DiO) was introduced into the formulations to measure the signal only from the liposomes themselves as little or no DiO was expected to be released. The liposomes were intravitreally injected into the center of the porcine eyes (Figure 1A). The detected signal was normalized to the initial signal measured at t = 0 (immediately after injection). A standard curve of DiO-liposomes dissolved in extracted vitreous fluid was obtained and a linear relationship (R^2^ = 0.97) was found between liposome concentration and fluorescence intensity, allowing normalization (Supplemental data, Figure S1C). The time-dependent signal decrease was measured for each formulation (Figure 1B–D) to estimate the time required to obtain a complete distribution of the liposomes in the pig eye (i.e., when baseline was reached). The area under the curves (AUC) depicted in Figure 1B–D were used as a single value to compare differences in the distribution dynamics of the liposomes (Figure 1E). Except for the cationic liposomes (Cat-Lp), all formulations were almost completely distributed in the vitreous after 24 h. The formulations without PEG (except Neu-Lp) diffused slower than the ones with PEG, and Cat-Lp diffused the slowest. Negatively charged liposomes with PEG (An-PEG-Lp) seemed to diffuse the fastest, although there was no statistically significant difference with the other PEGylated liposomes.

The diffusion coefficient of liposomes with different charges has been quantified for ex vivo vitreous dissected from porcine eyes [13]; however, fluorophotometry allowed us to measure liposome signal from completely intact eyes. Overall, a signal decrease over time was consistently observed, indicating that the system can be used to measure the spread of the injected formulation over time (Figure 1). In general, PEG-coated liposomes had a more rapid signal decrease in the vitreous compared with formulations without PEG, indicating a faster rate of diffusion. Cationic liposomes without PEG diffused slowest in the vitreous. However, PEGylation of this formulation decreased the AUC around 70%. This increased vitreal distribution of PEGylated cationic liposomes is consistent with previously known studies [7,17,31]. Somewhat surprising was the observation that anionic liposomes without PEG seemed to diffuse slower than neutral liposomes without PEG. This observation could be attributed to the inclusion of serine phospholipids (POPS) in the An-Lp formulation, which contain carboxylate in the head group. Although anionic, the carboxylate group could hinder the diffusion in the vitreous as it is a donor for hydrogen bonds and can interact with collagen fibers and hyaluronic acid [32]. Similarly, a study on the diffusion dynamics of polystyrene (PS) particles showed no statistical significant difference between cationic PS particles and carboxyl-coated PS particles in the porcine vitreous [33].

### 3.3. Histological Analysis

**Figure 2:**
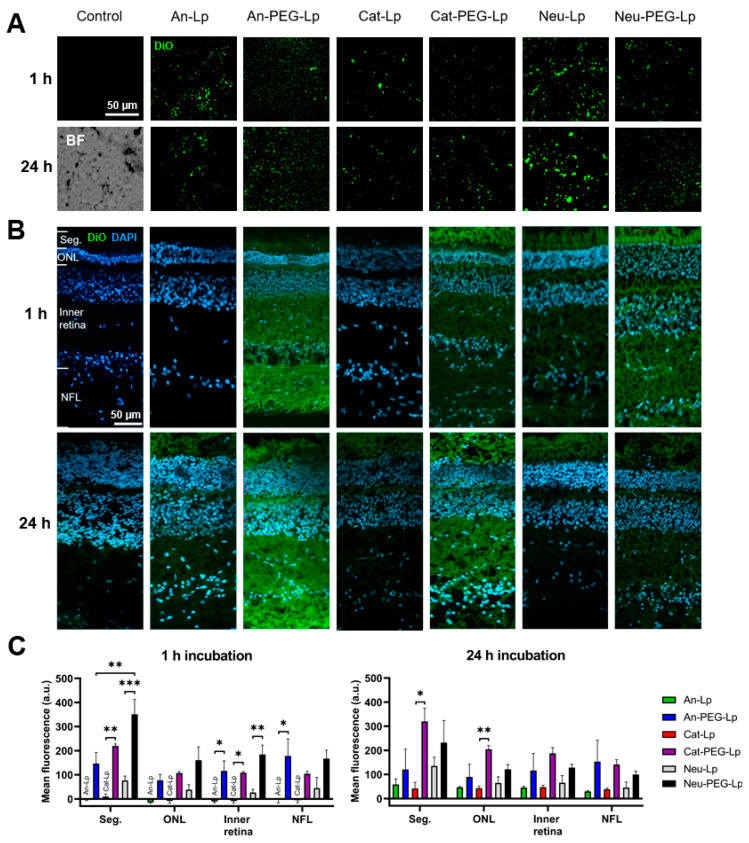
Histological analysis of ex vivo porcine vitreous and retinas from eyes that were fixed either 1 or 24 h after injection with liposome formulations. A green hydrophobic dye (DiO) was encapsulated in the formulations. (**A**) Images from vitreous sections after injection. Control = no liposomes injected. BF = bright-field image showing the structure of the vitreous. (**B**) Retina sections after injection. To analyze the retinal distribution of the fluorescent dye, the fluorescent intensity of different layers in the retina was quantified on the microscope software. The layers are depicted in the first image in the top right and are: photoreceptor inner and outer segments (Seg.), outer nuclear layer (ONL), inner retina (from the ganglion cell layer to the outer plexiform layer), and the nerve fiber layer (NFL). (**C**) Quantification of average fluorescent signal from retinal layers after 1 or 24 h post-injection into ex vivo porcine eyes. Control sections were used to obtain the background signal, which was subtracted from the other conditions. Results represent mean ± SEM for *n* = 3. * = *p* < 0.05, ** = *p* < 0.01, *** *p* < 0.001. Statistical analysis: One-way AVONA with Tukey’s post-hoc test.

The direct transport of liposomes to the retina is an important consideration when delivering hydrophobic compounds or macromolecules with an intracellular target (like genetic material). To determine the retinal absorption of DiO-labelled liposomes, histological sections were obtained from porcine eyes, which were fixed either 1 or 24 h post-injection (Figure 2). The 24 h time-point was chosen as most formulations seemed to have spread evenly in the vitreous (Figure 1B–D). Sections from the porcine vitreous (Figure 2A) and retina (Figure 2B) were analyzed for DiO signal. To assess the retinal distribution, the average fluorescent intensity from the retinal layers was measured using the acquisition software (Figure 2C).

On sections of the vitreous, the liposomes can be observed as green dots. In general, vitreous distribution seemed to be more even for PEGylated formulations, while more aggregations could be seen for non-PEGylated liposomes. The An-PEG-Lp formulation expressed the lowest degree of aggregation (smallest dots), while the more positively charged formulations (Cat-Lp and Cat-PEG-Lp) appeared to aggregate more in the vitreous. Although slightly anionic, the Neu-Lp formulation also showed a high degree of aggregation. This indicates that PEG is an important factor in the stability of liposomes in the vitreous.

From the analysis of the DiO signal in the retina, it is clear that charge and PEGylation affect the retinal uptake. All PEGylated formulations seemed to be taken up earlier when compared to non-PEGylated liposomes. The positively charged and PEGylated formulations (Cat-PEG-Lp and Neu-PEG-Lp) showed the highest uptake, with the highest signal coming from the photoreceptor segments. However, the Cat-Lp formulation, while showing a similar ζ-potential (Table 2), did not interact strongly with the retina, during the first 24 h, likely because the vitreous distribution was slower (Figure 1). Similarly, the An-Lp formulation diffused slowly in the vitreous, resulting in no signal at the retina after 1 h. Its PEGylated counter-part (An-PEG-Lp), on the other hand, showed high early-state absorption. 

### 3.4. Pharmacokinetic Evaluation

**Figure 3:**
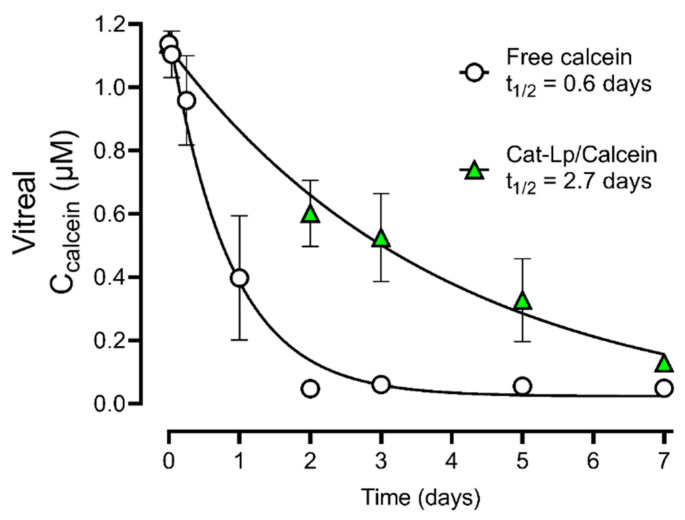
Estimation of the vitreal half-life (t_1/2_) of free calcein and calcein encapsulated in cationic liposomes (Cat-Lp/Calcein) after intravitreal injection into ex vivo porcine eyes. Vitreal calcein concentration was obtained from ocular fluorophotometry. The data shows a 4.5-fold increase in vitreal half-life when calcein was encapsulated. Data points were fitted to a one-phase exponential decay model (R^2^ = 0.92 for calcein and R^2^ = 0.72 for Cat-Lp/Calcein) calculated with GraphPad Prism 8 and represent mean ± SEM, for *n* = 3 (calcein) and *n* = 6 (Cat-Lp/Calcein).

We then determined whether the ex vivo porcine eyes could be used to estimate the pharmacokinetic parameters of a hydrophilic fluorophore, calcein, and to what extent liposome encapsulation would improve the pharmacokinetics. Calcein was chosen as it is released slowly from the liposomes [31]. The Cat-Lp formulation showed the slowest vitreous diffusion (Figure 1) and was thus expected to provide the longest retention time. When calcein was encapsulated in this liposome formulation, we observed a 4.5-fold increase in the vitreous half-life (Figure 3). To accurately evaluate the pharmacokinetic profiles with fluorophotometry, a homogenous distribution along the visual axis is required [34], i.e., an even signal throughout the vitreous. For free calcein, such a distribution was observed already after 1 h. For calcein encapsulated in Cat-Lp, it was only observed after more than 2 days.

### 3.5. Retina Viability Assay

**Figure 4:**
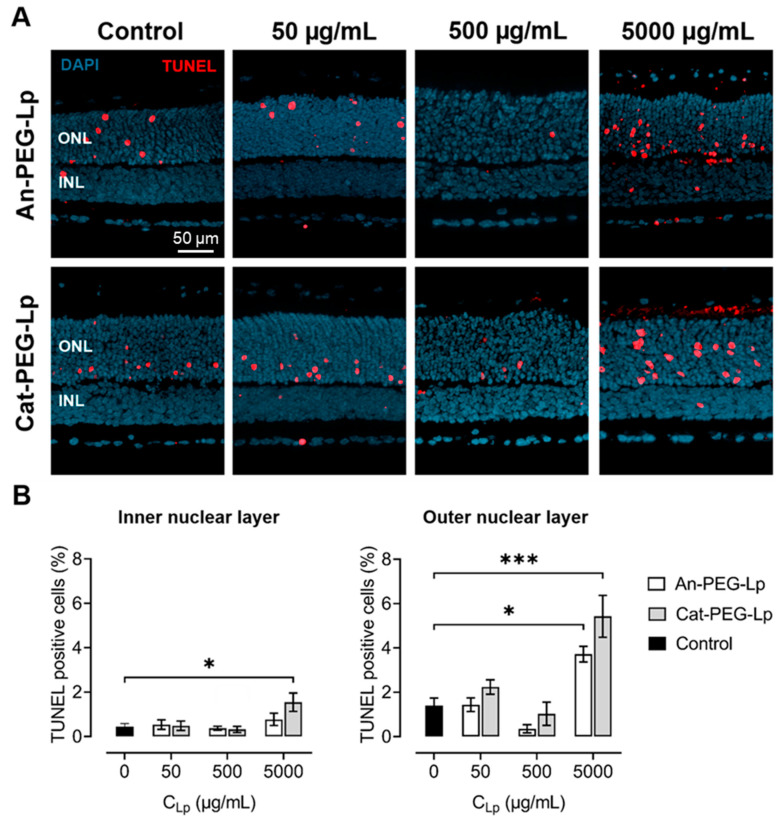
Retina viability assay. (**A**) TUNEL assay (red) was used to evaluate death of retinal cells from the inner nuclear layer (INL) and the outer nuclear layer (ONL) of retinal explant cultures after addition of anionic (An-PEG-Lp) and cationic (Cat-PEG-Lp) PEGylated liposomes at incremental concentrations. Control = no liposomes added. DAPI (blue) was used as nuclear counterstain. (**B**) Quantification of dying cells expressed as the percentage of TUNEL positive cells in the tissue sections. Results represent mean ± SEM for *n* = 3–6. * = *p* < 0.05, *** *p* < 0.001. Statistical analysis: One-way ANOVA with Dunnett’s post-hoc test.

The toxicity of the formulations to retinal cells was evaluated using a well-established organotypic retinal explant culture system [27]. As PEGylated liposomes were shown to reach the retina in the porcine eye model to a greater extend, formulations without PEG were not tested on murine retina. In addition, as the cationic liposomes included the lipid DOTAP, which has been shown to exhibit some cytotoxicity to HeLa cells [35], the formulations Cat-PEG-Lp and An-PEG-Lp were chosen for the analysis, to assess whether the addition of cationic lipids would cause more cell death to retinal cultures. The amount of dying retinal cells were quantified using the TUNEL assay (Figure 4). We found that the formulations were only toxic at high concentrations (5 mg/mL), with cationic liposomes causing more cell death than anionic liposomes. This effect was seen in cells of the inner nuclear layer, composed predominantly of second order neurons, and was even more evident in the outer nuclear layer, where the photoreceptors reside.

## 4. Discussion

In this study, we provide a new framework for testing the ocular distribution and retention of ocular drug delivery systems using ex vivo porcine eyes as a model. The ex vivo system along with ocular fluorophotometry and histology could give an early-state assessment of the behavior of liposomal drug delivery systems, in terms of distribution time, tissue interactions, and estimating relevant pharmacokinetic parameters of a model drug. The differences of the tested liposomes are summarized in Table 3. Interestingly, the PEG-coated formulations reach the retina faster than the non-PEG-coated counterparts, which could be accounted for by the vitreous diffusion. Although the An-PEG-Lp formulation diffuses the fastest, it does not absorb on the retina faster than the PEG-coated and positively charged liposomes, illustrating that these liposomes could be better suited for direct retinal transport. On the other hand, a possible limitation of positively charged liposomes could be the higher degree of toxicity.

Apart from a comparative analysis of different liposomes, non-invasive ocular fluorophotometry also allowed us to estimate the pharmacokinetics of a small hydrophilic fluorophore (calcein) in the intact eyeball (Figure 3). The use of fluorophotometry to assess ocular pharmacokinetics has previously been established [34,36]. For ex vivo porcine eyes, degeneration of the tissue limits long-term analysis. After day five, the vitreous became more and more liquefied, which could be observed by deflation of the eyeball. This happened even under sterile conditions. Liquefaction has been associated with increased flow, which would increase vitreous drug clearance [18]. To mitigate the issue in future experiments, medium-perfused ex vivo eyes have been used for a duration of up to 14 days [37], and could potentially keep the tissue fresh for longer. From the histological porcine retina sections (Figure 3), it appears that the retina is degenerating, which is worse after 24 h than after 1 h. This is obvious when comparing the structure with cultured retinas from mice (Figure 4). 

Viability of the tissue in the ex vivo model may influence the pharmacokinetic data obtained in the current study. Active transport processes and cell uptake of liposomes may decrease over time. Surprisingly, however, the vitreous half-life of calcein that was obtained ex vivo here (0.6 days) agrees well with that of a recent in vivo rabbit study, which measured the intravitreal calcein pharmacokinetics [38]. Although the rabbit vitreous is smaller than the porcine, it is generally understood that the vitreous diffusion is not the rate-limiting step for retention of small molecules [3]. Our data suggests that at least the short-term pharmacokinetics may be comparable to the in vivo situation. In the aforementioned study, the half-life of calcein loaded in liposomes was extended to seven days, which is more than in our case (2.7 days). The composition of the liposomes in the two studies were different and the release of calcein from the liposomes used in the in vivo rabbit study is expected to be slower. In the latter study lipids with a high transition temperature were used (hydrogenated L-α-phosphatidylcholine), while lipids with a low transition temperature (POPC) were used in the current study. Calcein is released from POPC liposomes approximately three times quicker than from the hydrogenated counterpart, as shown by Maherani et al. [31]. This would thus affect the vitreous half-life of calcein.

The diffusion and vitreal half-life of intravitreally administered drugs are not only very dependent on the liposome system used, but also on the properties of the drug. For instance, liposomal 5-Fluorouridine 5′MP was reported to have a half-life of 5.2 days while for liposomal Gentamicin this was less than two days [4]. With the ex vivo, non-invasive imaging approached used here, a comparable analysis between the encapsulated and non-encapsulated compound is possible. We obtained an extension of 4.5 times the intravitreal half-life when calcein was encapsulated in liposomes, which is within the range of what would be expected from in vivo studies. The previously mentioned liposomal Gentamicin system only extended the half-life by a factor of 1.2, and for a liposomal Amikacin system [11], the half-life was extended by 1.8. However, liposomal 5-Fluorouridine 5′MP saw a 27-fold increase in intravitreal half-life. 

The limitation of pharmacokinetic evaluation using fluorophotometry is that only fluorescent markers can be detected, i.e., only the retention of fluorescent drugs or fluorescently-labelled drugs can be tested. Labelling of the drug molecule has the disadvantage that it changes the chemistry and potentially the release profile of the drug. The major advantage of fluorophotometry, however, is that intact eyes can be used, and the only intervention required is the injection. Different routes of applications (e.g., intravitreal, topical, subconjunctival) can be compared. The real-time pharmacokinetics can be measured, which is especially relevant for in vivo studies, since there is no need for sampling the vitreous or aqueous humor fluid or sacrificing the animal for every measurement. Even further, the very same methodology may be used to non-invasively study the pharmacokinetics of drug delivery systems in clinical trials. In other words, fluorophotometry may provide for a direct comparison between ex vivo models, preclinical in vivo models, and clinical trials, using the same methodology and instrumentation.

In the ex vivo porcine eye model, the biodistribution of the formulations is another parameter, which can be tested and give insight into the potential of a given drug delivery system. Liposome-mediated delivery of macromolecules such as peptides and genetic materials (e.g., plasmids [39] and siRNA [40]), which have an intracellular target, requires direct transport to the cells. We have shown that the ex vivo model can answer whether the delivery system is able to reach the target of interest (e.g., the retina) by passive diffusion and offers an estimate about how much time would be required. It is, however, questionable whether the retinal cells of the ex vivo model take up the drug delivery system through active processes. Active cell uptake would have to be confirmed with cell or tissue cultures.

According to our results, PEGylated liposomes diffused to the retina within 1 h (Figure 2), and could be detected in the outer retina. Previously, PEGylated liposomes have been shown to improve retinal transport compared with non-PEGylated liposomes [41]. Since cationic liposomes interact stronger with the retinal tissue, we obtained a higher signal for these liposomes (Cat-PEG-Lp and Neu-PEG-Lp), especially in the deeper parts of the retina (outer nuclear layer and photoreceptor segments). Whether the DiO signal coming from the outer retina is from intact liposomes or unbound DiO transported throughout the retina, possibly in extracellular vesicles, is not clear from these tests. Nevertheless, the results show that a hydrophobic model drug can be transported throughout the retina. We tried to analyze the histology of eyes injected with calcein-loaded liposomes, but no signal was detected (data not shown). This could be due to substantial calcein release and elimination caused by the histological preparation procedure, which include freezing and washing steps. DiO, on the contrary, is not expected to be released from liposomes dissolved in the vitreous.

For ocular drug delivery systems with a retinal target, there is a strict requirement of safety, since retinal cells are predominantly post-mitotic cells that cannot be replaced once lost. We found that the retina can safely tolerate up to 500 µg/mL of liposomal formulations, even for positively charged liposomes. Although only two formulations were tested here, similar toxicity profiles would be expected for the other formulations investigated in this study. In retinal cultures, more dying cells were observed in the outer nuclear layer, where the cell bodies of photoreceptors reside, compared to the inner nuclear layer. However, this is not necessarily an effect of the formulation, since the control condition, where cell death is primarily caused by the explanation procedure, showed a similar trend. The observation that in murine explant cultures photoreceptors are more sensitive than second order neurons in the inner retina is consistent with previous data [30,42].

In summary, we provide a first systematic overview of the intravitreal behavior of different liposomal formulations in the ex vivo porcine eye. This approach can supply important information about ocular drug delivery systems, especially regarding their intravitreal pharmacokinetics and biodistribution, without requiring the use of experimental animals. Importantly, the non-invasive imaging approach used here can be directly transferred to corresponding pre-clinical in vivo studies and even clinical studies, allowing a direct comparison of measurement parameters across different systems and species. 

## Figures and Tables

**Table 1:** Molar percentages of lipid components in the liposomes.

Formulation	POPC	Cholesterol	DOTAP	POPS	DSPE-PEG_2000_
An-Lp	44.4%	33.3%		22.2%	
An-PEG-Lp	63.3%	31.7%			5%
Cat-Lp	44.4%	33.3%	22.2%		
Cat-PEG-Lp	42.2%	31.7%	21.1%		5%
Neu-Lp	66.6%	33.3%			
Neu-PEG-Lp	58.3%	31.7%	5%		5%

An: anionic, Cat: cationic, Neu: neutral, PEG: polyethylene glycol, Lp = liposome.

**Table 2:** Characterization of liposomes. Data are presented as mean ± SD for *n* = 3.

Formulation	φ (nm)	PDI	ζ-potential (mV)
An-Lp	131 ± 2	0.046 ± 0.00	−55 ± 3
An-PEG-Lp	127 ± 2	0.051 ± 0.01	−34 ± 3
Cat-Lp	135 ± 5	0.078 ± 0.01	42 ± 2
Cat-PEG-Lp	129 ± 2	0.054 ± 0.02	41 ± 1
Neu-Lp	163 ± 4	0.166 ± 0.03	−12 ± 1
Neu-PEG-Lp	154 ± 17	0.164 ± 0.03	29 ± 2

PDI: polydispersity index.

**Table 3:** Overview of relevant properties of the investigated liposomes.

Formulation	Vitreous Diffusion	Vitreous Retention	Retinal Absorption	Retinal Toxicity
An-Lp	Slow	High	Very slow	
An-PEG-Lp	Very fast	Very low	Fast	Low
Cat-Lp	Very slow	Very high	Very slow	
Cat-PEG-Lp	Fast	Low	Very fast	Intermediate
Neu-Lp	Fast	Low	Intermediate	
Neu-PEG-Lp	Fast	Low	Very fast	

## Data Availability

Not applicable.

## Supplementary Materials

The following are available online at https://www.mdpi.com/article/10.3390/pharmaceutics13071013/s1, Figure S1: standard curves of calcein and DiO.
