# Supplementary material for: Investigating Ex Vivo Animal Models to Test the Performance of Intravitreal Liposomal Drug Delivery Systems
Source: Pharmaceutics. 2021 Jul 2;13(7):1013. doi: 10.3390/pharmaceutics13071013 (PMC8309192; doi:10.3390/pharmaceutics13071013)
Supplement: Supplementary file 1 [file pharmaceutics-13-01013-s001.zip › pharmaceutics-1263511-supplementary.pdf]

pharmaceutics

Supplementary Materials: Investigating Ex Vivo Animal Models to Test the Performance of Intravitreal Liposomal Drug Delivery Systems

Gustav Christensen, Leon Barut, Dileep Urimi, Nicolaas Schipper, and François Paquet-Durand

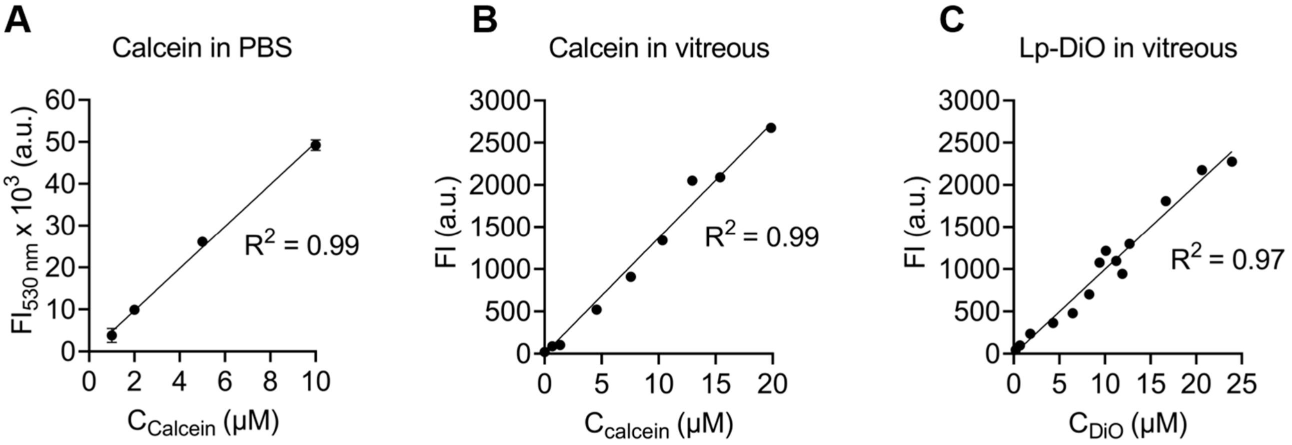

Figure S1. Standard curves of calcein and DiO. (**A**) Calcein in PBS, measured at 530 nm. (**B**) Calcein in extracted porcine vitreous, measured on an FM-2 Fluorotron Master. (**C**) DiO-loaded liposomes (Lp-DiO) in extracted porcine vitreous, measured on an FM-2 Fluorotron Master. All data points were fitted with linear regression using GraphPad Prism 8. All p-values < 0.0001.
